# The prevalence and identity of *Chlamydia*-specific IgE in children with asthma and other chronic respiratory symptoms

**DOI:** 10.1186/1465-9921-13-32

**Published:** 2012-04-18

**Authors:** Katir K Patel, Erica Anderson, Paul S Salva, Wilmore C Webley

**Affiliations:** 1 Department of Microbiology, University of Massachusetts, Amherst, MA, USA; 2 Department of Pediatric Pulmonology, Baystate Medical Center, Springfield, MA, USA; 3 Department of Microbiology, University of Massachusetts, 639 North Pleasant Street, 203 Morrill IV N, Amherst, MA 01003, USA

**Keywords:** Asthma, Specific IgE, *Chlamydia*

## Abstract

**Background:**

Recent studies have confirmed the presence of viable *Chlamydia* in the bronchoalveolar lavage (BAL) fluid of pediatric patients with airway hyperresponsiveness. While specific IgG and IgM responses to *C. pneumoniae* are well described, the response and potential contribution of Ag-specific IgE are not known. The current study sought to determine if infection with *Chlamydia* triggers the production of pathogen-specific IgE in children with chronic respiratory diseases which might contribute to inflammation and pathology.

**Methods:**

We obtained BAL fluid and serum from pediatric respiratory disease patients who were generally unresponsive to corticosteroid treatment as well as sera from age-matched control patients who saw their doctor for wellness checkups. *Chlamydia*-specific IgE was isolated from BAL and serum samples and their specificity determined by Western blot techniques. The presence of *Chlamydia* was confirmed by species-specific PCR and BAL culture assays.

**Results:**

Chlamydial DNA was detected in the BAL fluid of 134/197 (68%) patients. Total IgE increased with age until 15 years old and then decreased. *Chlamydia*-specific IgE was detected in the serum and/or BAL of 107/197 (54%) patients suffering from chronic respiratory disease, but in none of the 35 healthy control sera (*p* < 0.0001). Of the 74 BAL culture-positive patients, 68 (91.9%, *p* = 0.0001) tested positive for *Chlamydia*-specific IgE. Asthmatic patients had significantly higher IgE levels compared to non-asthmatics (*p* = 0.0001). Patients who were positive for *Chlamydia* DNA or culture had significantly higher levels of serum IgE compared to negative patients (*p* = 0.0071 and *p* = 0.0001 respectively). Only 6 chlamydial antigens induced *Chlamydia*-specific IgE and patients with *C. pneumoniae-*specific IgE had significantly greater levels of total IgE compared to *C. pneumoniae*-specific IgE negative ones (*p* = 0.0001).

**Conclusions:**

IgE antibodies play a central role in allergic inflammation; therefore production of *Chlamydia*-specific IgE may prove significant in the exacerbation of chronic, allergic airway diseases, thus highlighting a direct role for *Chlamydia* in asthma pathogenesis.

## Introduction

Inflammation of the airways is the most common finding in all asthma patients and today, most asthma experts consider airway inflammation a central feature of asthma pathogenesis [1,2]. The genetic predisposition to asthma development has been well recognized and the IgE-mediated response to common aero-allergens represents the most common form of the disease in childhood and early adulthood [3]. Published evidence strongly suggests a relationship between microbes and asthma [4]. It has long been known that the most potent triggers of wheezing are mediated by viruses, however, we do not understand the mechanisms involved in how they contribute to disease onset and progression. Even less is known about the relationship between asthma and bacteria. Recent studies confirm that bacterial respiratory infections are frequently associated with increased airway obstruction in patients with bronchial asthma [3]. While the hygiene hypothesis predicts that infections in early life by non-pathogenic microbes should protect against asthma and atopy [5], there is increasing evidence that certain chronic pathogenic infections might also promote airway hyperresponsiveness and asthma development or exacerbation [6-8].

Approximately 20% of wheezing children have serological evidence of an immune response to *Streptococcus pneumoniae*, *Haemophilus influenzae* and *Moraxella catarralis*[9,10]*.* More recently, *Mycoplasma pneumoniae* and *Chlamydia pneumoniae* have been identified in 5–25% of children with asthma exacerbations [11-13]. Hahn et al also reported a significant improvement in overall asthma symptoms at treatment completion which persisted for 3 months despite withdrawal of azithromycin in an adult population [14,15].

Earlier studies by Welliver *et al.*[16] reported IgE responses to RSV in children with bronchiolitis and showed that this IgE response was related to recurrent wheeze, but not to lung function and allergic sensitization at an early age. This suggests that IgE antibodies to RSV occur independently of atopy and may be indicative of an ongoing asthmatic process. Specific IgE to bacterial colonizers has also been reported. *H. influenzae* and *S. pneumoniae* specific IgE antibodies have been found in the serum of approximately a third of atopic children and asthmatic adults, however, they were all related to a subject’s atopic status [17]. The implication here is that IgE has a complex relationship with asthma that might not be dependent on the specific allergens that are routinely assayed for [18].

In the current study we examined the BAL fluid and serum of a large cohort of children with chronic respiratory disease for the presence of *Chlamydia*-specific IgE antibodies. We hypothesize here that the presence of *Chlamydia*-specific IgE antibodies explains, at least in part, the mechanism of chlamydial involvement in either initiating or exacerbating chronic allergic airway disease.

## Methods

### Patients and samples

Serum and BAL samples were analyzed from 197 patients between the ages of 0–20 who were patients at Baystate Medical Center. BAL samples were originally obtained from patients for diagnostic or treatment purposes. Children were considered for bronchoscopy only after a thorough, non-invasive evaluation did not yield a definitive diagnosis; symptoms were not improving with time; aggressive medical management had not been successful and the child was truly compromised i.e., was missing school, could not participate in physical activity and had frequent symptoms. Airway reactivity was monitored by spirometry in children capable of performing the test as well as clinically, by frequency and severity of symptoms and response to therapy. In children not capable of performing spirometry, clinical characteristics were assessed.

We also obtained 35 age and gender-matched control patient sera as residual samples from patients undergoing wellness check for school records or general checkup at the University of Massachusetts Health Services complex over a similar period of time as the patient samples. We obtained IRB approval and patient consent to collect and use these residual samples for research from the Baystate Medical Center and University of Massachusetts Amherst IRB. In addition, a subset of 20 (of the 197) respiratory disease patients presented with physical airway anomalies including most commonly, tracheal bronchus, obstruction by a foreign object, laryngomalacia, tracheomalacia, and bronchomalacia with abnormal cough. These were considered non-inflammatory controls when compared with asthmatics and patients with pneumonia and bronchitis.

### *Chlamydia* detection in patient samples

Genomic DNA was isolated from BAL samples and PCR detection of chlamydial DNA was performed in the same manner as previously described for all samples in this cohort [19]. All BAL samples were also analyzed by tissue culture techniques to determine *Chlamydia* viability as previously reported [12,19]. DNA was also isolated from control serum samples in a similar manner as the BAL and evaluated using the same primers.

### Total IgE evaluation

Total IgE was evaluated using the Elecsys IgE kit (Roche Diagnostics, Indianapolis, IN), with the electrochemiluminescence immunoassay according to the manufacturer’s instructions. Plates were read on the Roche Elecsys 1020 analyzer which automatically calculated the IgE concentration of each sample based on a standard curve. Elevated IgE levels were determined based on the manufacturer’s recommended threshold by age range.

### Isolating serum and BAL IgE antibodies

Because the normal concentration for IgE in serum is approximately 0.0005 mg/ml, and is even less in BAL fluid, we utilized affinity beads in a similar manner as Kadooka et al [20] to isolate the IgE antibodies in order to ensure effective reaction of these antibodies with chlamydial antigens on our blots. We used recombinant Protein G sepharose gel (Sigma-Aldrich, St. Louis, MO) to adsorb IgG antibodies from the serum samples. Since recombinant protein G does not bind IgE antibodies [21,22], individual patient serum samples were added to the beads and allowed to bind with slow stirring overnight. The supernatant that was now ‘enriched’ for IgE antibodies was then removed and analyzed for the presence of *Chlamydia*-specific IgE antibodies.

To test for IgE in BAL samples, recombinant protein A beads (Sigma-Aldrich, St. Louis, MO) were utilized in the same manner as the protein G beads above. In this case the protein A beads were used to adsorb any antibodies detectable in the BAL fluid. IgE antibodies present in the BAL fluid were bound by the protein A beads and the bound antibodies were eluted using a pH 2.5 glycine solution followed by centrifugation to obtain the supernatant containing IgE and other antibodies if present. The eluted antibodies were pH balanced using Tris–HCl and then used as the primary antibody in the Western blot procedure. Because of the limited volume of some BAL fluid samples available and the low concentration of IgE in each sample, BAL samples were initially assessed in batches of 4 to 7 samples each.

### Western blot procedure

As previously reported, Western blot is a highly sensitive and efficient technique in detecting specific IgE [23-25]. *C. pneumoniae* (TW183) and *C. trachomatis* (serovar E) elementary bodies were purified by 20%–50% (vol/vol) Renografin gradient centrifugation as previously described [26] and normalized for protein content using the Bradford protein assay. Proteins were separated by electrophoresis on NuPage 4–12% Bis Tris gels (Invitrogen, Carlsbad, CA). Following electrophoresis the separated proteins were transferred to PVDF membranes, blocked and each well was cut into individual strips. Each strip was incubated with patient sample that had been processed with protein A or G beads overnight. After incubation, the strips were washed and a 1:500 dilution of AP-conjugated anti-human IgE, epsilon chain specific antibody (KPL, Gaithersburg, Maryland) was added to each strip for 2 hours. Strips were again washed and developed with a BCIP/NBT alkaline phosphatase substrate and reactions were stopped after several washes with ultrapure distilled water. Blot strips were analyzed for the presence and identity of *Chlamydia*-specific IgE bands. All samples were assayed for the presence of specific IgE using identical conditions including sample dilution.

### Cell counts

BAL cell counts and differentials were performed according to standard techniques in the hospital’s Hematology Clinical Laboratory at Baystate Medical Center (Springfield, MA) as previously reported [19,27]. Briefly, cell enumeration was performed manually using a cell counting chamber under phase microscopy with results expressed as “number of cell/mm^3^. BAL differential counts were performed using Wright stained cytospin preparations of BAL and examination was performed under oil-immersion microscopy (50X or 100X). Results were expressed on the basis of a 100 cell count survey. Inflammatory asthma sub-phenotypes were determined based on BAL neutrophil and eosinophil cells counts, as previously reported [28].

### Statistical analysis

All statistical analyses among the different groups of the patient population were performed using Microsoft Excel®. Statistical correlations were determined with Fisher’s exact test and Chi-square with Yates correction (SPSS 15.0 software). A *p*-value of 0.05 was considered significant and all tests were two-tailed.

## Results

### Characteristics of study patients

**Figure 1:**
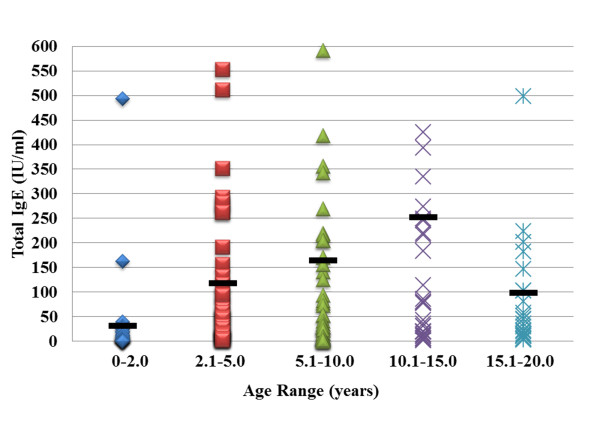
**Total Serum IgE levels generally increased with age.** Assessment of total serum IgE levels showed a general increase with age up to age 15.0 years old (40 IU/ml to 250 IU/ml). The levels then decreased to 100IU/ml. Dark lines represent the average IgE level for each age range.

We obtained both BAL fluid and serum from 197 pediatric respiratory disease patients, average age 7.9 years old, who presented to a pediatric pulmonary practice in Springfield, MA with chronic respiratory disease and were generally unresponsive to corticosteroid treatment. The patient demographic (Table 1), shows that 143/197 (73%) were diagnosed with asthma and that these asthmatics were predominantly between the ages of 2 to 10 years old. We utilized a healthy control group of 35 patients, average age 5.8 years, 21 males/14 females who saw their doctors for wellness checks. Total serum IgE generally increased with age up to 15.0 years old (40 IU/ml to 250 IU/ml; see Figure 1). The levels decreased significantly in the 15.1 to 20.0 year old age group.

**Table 1:** Patient demographics of the pediatric patient cohort

**Description**	Asthma (*percent within asthma)	Non-asthma (*percent within non-asthma)	**Total**
Diagnosis	143 (72.6%)	54 (27.4%)	197
Average age	8.3	7.1	7.9
Age range			
0-2.0	14 (9.8%)	16 (29.6%)	30
2.1-5.0	38 (26.6%)	13 (24.1%)	51
5.1-10.0	41 (28.7%)	8 (14.8%)	49
10.1-15.0	29 (20.3%)	9 (16.7%)	38
15.1-20.0	21 (14.6%)	8 (14.8%)	29
Gender			
Female	65 (45.5%)	25 (46.3%)	90
Male	78 (54.5%)	29 (53.7%)	107
Ethnicity			
White	107 (74.8%)	43 (79.6%)	150
Black	7 (4.9%)	4 (7.4%)	11
Hispanic	29 (20.3%)	6 (11.1%)	35
Asian	0 (0.0%)	1 (1.9%)	1
BMTH			
Yes	118 (82.5%)	17 (31.5%)	135
No	10 (7.0%)	18 (33.3%)	28
Not Done	15 (10.5%)	19 (35.2%)	34

Most of the patients in this cohort were asthmatics (72.6%) with an average age of 8.3 years old (7.9 years old for entire cohort). The majority of asthma patients were between ages 2 to 10 years old and basement membrane thickening (BMTH) was significantly correlated with asthma diagnosis.

### Prevalence of *Chlamydia* in patient cohort

Polymerase chain reaction (PCR) was utilized to determine if *C. trachomatis* and/or *C. pneumoniae* organisms were present in patient BAL samples. A total of 134/197 (68%) patient samples were positive for chlamydial DNA. Species-specific PCR revealed that 65 samples were positive for *C. pneumoniae* DNA only, 34 were positive for *C. trachomatis* DNA only and 35 patients harbored both *C. trachomatis* and *C. pneumoniae* DNA. In order to determine if the organisms detected by PCR were viable, all BAL samples were subjected to a modified tissue culture technique as previously described. The data showed that 74/197 (38%) patients assayed were culture positive for *Chlamydia*. Importantly, 62/74 (84%, (Fisher’s exact test *p* < 0.0001) culture positive patients were diagnosed with chronic, severe asthma according to GINA guidelines [29]. Analyses of the control patient sera revealed that 7/35 (20%) were positive for the presence of chlamydial DNA.

### Total IgE antibody serum levels

**Figure 2:**
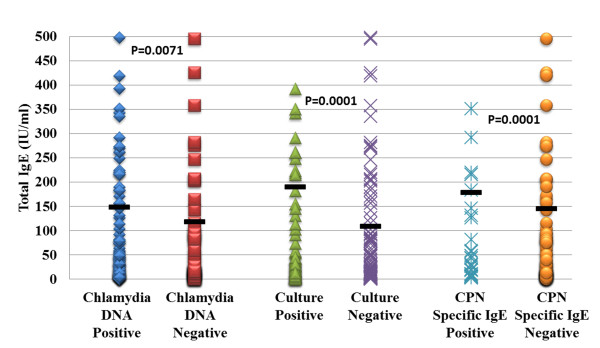
**Presence of *Chlamydia *organisms is associated with higher levels of serum IgE.** While the average total serum IgE levels in this patient cohort was not significantly elevated (138 IU/ml), IgE levels were significantly more elevated in patients who were either *Chlamydia* DNA positive or who had viable organisms as determined by culture. Patients with *C. pneumoniae*-specific IgE also had an average total IgE level that was higher than their *C. pneumoniae*-negative counterparts. Horizontal dark bars represent the average IgE level and each shape in the respective columns represents a single patient.

Data analysis revealed that 65/197 (33%) patients had elevated serum IgE levels, while 132/197 (67%) had normal IgE levels (Fisher’s exact T-test *p* < 0.0001). Patients who were positive for *Chlamydia* by DNA or culture had significantly higher levels of total serum IgE compared to IgE negative patients (*p* = 0.0071 and *p* = 0.0001 respectively; Figure 1). Patients who tested positive for *C. pneumoniae* specific IgE antibodies also had an average IgE level that was higher than the specific IgE negative cohort. Total serum IgE generally increased with age up to 15.0 years old (40 IU/ml to 250 IU/ml; see Figure 2). The levels decreased significantly in the 15.1 to 20.0 year old age group.

### *Chlamydia*-specific IgE antibody is present in the BAL and serum of infected patients

**Figure 3:**
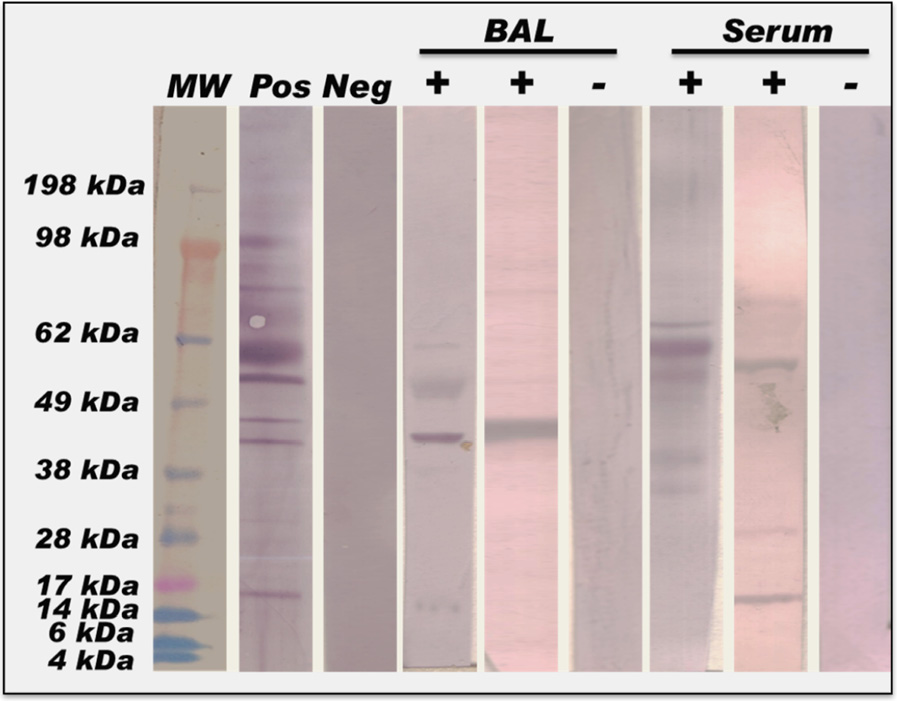
**Western blot of *Chlamydia*-specific IgE. ** The blots depict representative patient BAL and serum samples that were positive for the presence of *Chlamydia*-specific IgE. Band specificities were determined by a *Chlamydia*-positive control and the *Chlamydia* protein data-base was used to confirm the corresponding protein. Note that of all the chlamydial proteins seen on the control strip stained with a polyclonal antibody solution, only a finite number are recognized by specific IgE antibodies.

In light of the increased prevalence of chlamydial organisms in the BAL fluid of patients in this cohort, patient serum and BAL samples were analyzed to determine the response of the host to the organism in each case and to determine if there is pathology associated with its presence. We therefore developed a Western blot assay to determine the presence, prevalence and identity of *Chlamydia*-specific IgE in both the serum and BAL fluid from each patient. Since BAL samples were assessed in batches of 4 to 7 samples each (because of limited sample volume), we cannot ascertain the exact percentage of patients with *Chlamydia*-specific IgE in their BAL fluid. However, qualitatively, BAL fluid samples from this patient cohort contained *Chlamydia*-specific IgE antibodies (Figure 3). To our knowledge, this is the first report of pathogen-specific IgE in human BAL fluid.

*Chlamydia*-specific IgE was also detected in the sera of 107/197 (54%) patients suffering from chronic respiratory disease but none of the healthy control patient sera (0/35, *p* < 0.0001). In addition, a subset of 20 patient samples from our respiratory disease cohort who were diagnosed with structural anomalies rather than inflammatory airway disease, were also assessed for the prevalence of *Chlamydia*-specific IgE. The data shows that 1/20 (5%) had *Chlamydia*-specific IgE in their sera compared to the inflammatory patient cohort (Table 2). Assessment to determine the prevalence of *Chlamydia*-specific IgE antibodies in the BAL culture-positive patient cohort showed that 68/74 (91.9%, *p* = 0.0001) tested positive for *Chlamydia*-specific IgE.

**Table 2:** The Prevalence of *Chlamydia*-specific IgE in Patient Sera

	**Respiratory Disease**	**Non-Inflammatory Disease**	**P-values**	**Healthy Controls**	**P-values**
**Number of Patients**	177	20		35	
**Serum IgE, No. (%)**					
**Positive**	97 (55%)	1 (5%)	0.0030*	0 (0%)	0.0001*
**Negative**	80 (45%)	19 (95%)		35 (100%)	
**Chlamydial DNA, No. (%)**					
**Positive**	124 (70%)	10 (50%)	0.4417	7 (20%)	0.0208*
**Negative**	53 (30%)	10 (50%)		28 (80%)	

Fifty five percent of patients with inflammatory respiratory disease had *Chlamydia*-specific IgE antibodies in their sera compared to 5% of the 20 patients with structural anomalies and 0% of healthy controls (*p* = 0.0030 and 0.0001 respectively. 70% of the patients diagnosed with respiratory disease also tested positive for chlamydial DNA, whereas only 20% of healthy controls tested positive for chlamydial DNA (*p* = 0.0208).

**Figure 4:**
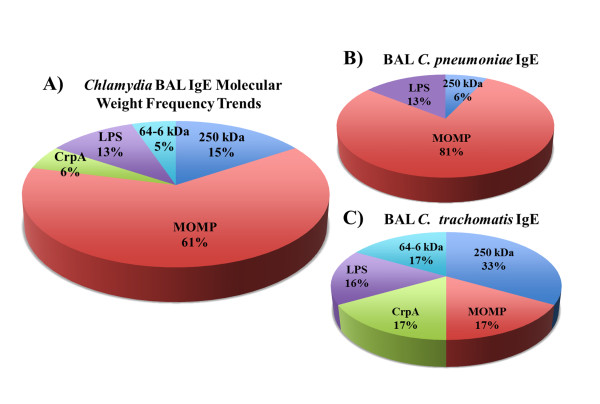
***Chlamydia *protein molecular weight frequency trends in BAL fluid. ** Chart A shows the overall molecular weight of proteins recognized by *Chlamydia*-specific IgE antibodies from patient BAL samples. These are for *C. trachomatis* and *C. pneumoniae* specific antibodies combined. The major outer membrane protein (MOMP) is recognized 61% of the time by these positive patient sera. However, chart B clearly shows that the MOMP from *C. pneumoniae* organisms were more likely to induce IgE antibodies than *C. trachomatis* MOMP (Chart C). The hypothetical 250KDa protein was the most frequently recognized *C. trachomatis* protein in BAL.

Both *C. trachomatis* and *C. pneumoniae*-specific IgE antibodies were found in the BAL fluid (Figure 4). Importantly, although *Chlamydia* has 800–1000 coding genes, only 6 chlamydial antigens appear to induce pathogen-specific IgE responses in this patient cohort and the frequency of these bands differ significantly between *C. trachomatis* and *C. pneumoniae* (Figure 4B and 4C). In fact, most of the patient samples contained IgE antibodies to a single protein band on the blots (see Figure 3).

Overall, 61% of the *Chlamydia*-specific IgE antibodies in BAL fluid recognized the chlamydial major outer membrane protein (MOMP). This was generally true however, for *C. pneumoniae* IgE antibodies, where 81% of the positive patients had IgE that recognized MOMP, compared to only 17% for *C. trachomatis* MOMP. Thirty three percent of BAL fluid positive patient samples displayed IgE antibodies against an un-annotated 250kDa *C. trachomatis* protein.

**Figure 5:**
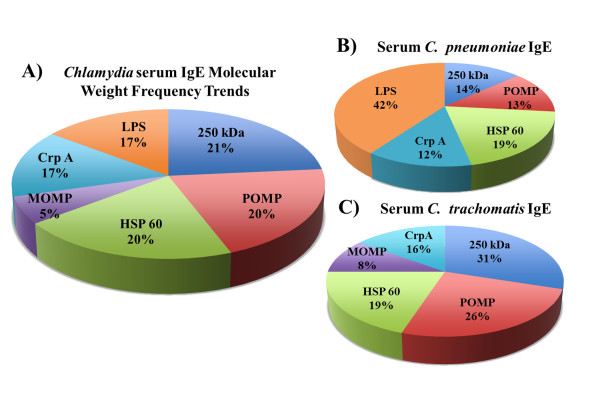
***Chlamydia *protein molecular weight frequency trends in Serum. ** Unlike the BAL fluid proteins in Figure 2, serum IgE antibodies against MOMP only appeared in 5% of the patients tested, while the other 5 antigens appeared at similar frequency to that of the BAL (Chart A). Closer analysis shows that while serum IgE antibodies recognized *C. pneumoniae* LPS in 42% of positive samples (Chart B), no *C. trachomatis* LPS was recognized by these samples (Chart C). Although MOMP is the major antigen on the chlamydial EB surface, *C. pneumoniae* MOMP did not induce serum IgE production in this study.

Assessment of individual serum samples for the presence of *Chlamydia*-specific IgE antibodies revealed bands on Western blot ranging in molecular weight from 6kDa to 250kDa (Figure 5A). As was true for the IgE antibodies in the BAL fluid, the identity of serum IgE antibodies produced in response to *C. trachomatis* were significantly different from *C. pneumoniae* – specific antibodies (Figure 5B and C). Antigen frequency analysis revealed that 42% of IgE positive serum samples contained IgE antibodies that recognized *C. pneumoniae* chlamydial lipopolysaccharide (cLPS). *C. trachomatis* - induced cLPS IgE antibodies were not detected in any serum sample. *C. pneumoniae* did not induce MOMP-specific IgE in serum, while 8% of IgE positive-patients had antibodies that recognized *C. trachomatis* MOMP (Figure 5C). There were also significant differences in the production of *Chlamydia*-specific IgE in the serum versus BAL fluid. Chlamydial heat shock protein 60 (Hsp 60 or GroES) and the polymorphic outer membrane protein (POMP) did not induce IgE antibodies in the BAL fluid, however, they each accounted for the *Chlamydia*-specific IgE found in 20% of positive patient sera assayed. The frequency of MOMP-induced IgE in the BAL fluid was 61%, while the frequency in serum was only 5%, demonstrating possible site-specific differential cytokine and lymphocyte responses or antibody release (see Figures 4A and 5A).

### Inflammatory asthma phenotype and serum IgE concentrations

**Figure 6:**
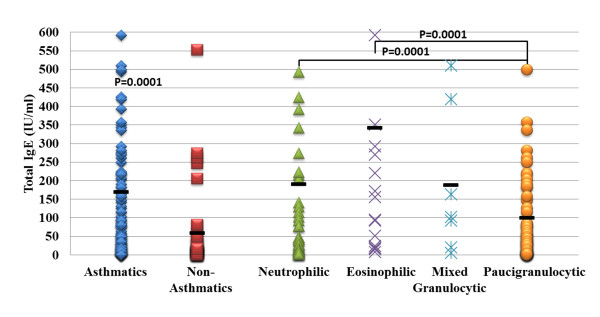
**Asthma phenotypes and total serum IgE levels.** Asthmatic patients had significantly higher total IgE levels compared to non-asthmatic patients. Inflammatory asthma phenotypes determined by BAL cell counts revealed that eosinophilic asthmatics had higher IgE levels compared to non-eosinophilic phenotypes (neutrophilic, paucigranulocytic and mixed granulocytic patients).

Eosinophilic inflammation, characterized by high levels of eosinophils, atopy and elevated serum IgE concentration, has long been considered one of the most distinctive pathological hallmarks of asthma [30]. However, it has recently been recognized that airway eosinophilia is not a universal finding and a significant subset of patients with refractory asthma display a non-eosinophilic phenotype. BAL cell counts revealed that 115/143 (80%) asthma patients in this cohort presented with a non-eosinophilic inflammatory asthma phenotype. Overall, the majority of patients in this cohort 166/197 (84%) had normal BAL eosinophil counts. Asthmatic patients had a significantly higher total IgE level compared to non-asthmatic patients and patients with an eosinophilic asthma phenotype had significantly higher total serum IgE levels than other inflammatory asthma groups (*p* = 0.0001; see Figure 6).

## Discussion

The nature of the clinical association that exists between *Chlamydia* airway infection and subsequent development of asthma in young children remains controversial. In an effort to find discriminative serological or biological markers to differentiate between patients with infection-mediated airway responsiveness and those with other aeroallergen-specific disease, we utilized Western blot techniques to determine and compare the *Chlamydia*-specific IgE response in the sera of 197 pediatric patients with chronic, severe respiratory disease.

The development of pathogen-specific IgE antibodies is not unique to *Chlamydia* infection. Numerous reports have demonstrated production of specific IgE antibodies in response to infection by pathogens, including a variety of viruses [31-33] and Mycoplasma [34]. Emre *et al*, previously reported the presence of *C. pneumoniae*-specific IgE in a cohort of children with chronic respiratory disease, and suggested that production of specific IgE may be an underlying mechanism leading to reactive airway disease in some patients with *C. pneumoniae* infection [35]. With our larger cohort, our data is in line with this previous report and is strongly predictive of severe reactive airway disease and poor asthma outcome. To this end, while the role of pathogen-specific IgE antibodies has still not been fully determined, when studied, in most cases, the responses were associated with a poorer prognosis [33].

Approximately 68% of the patients analyzed in the current study harbored chlamydial DNA and 54% produced detectable levels of specific IgE antibodies in response to chlamydial infection. Age matched, healthy controls had no detectable *Chlamydia*-specific IgE in their serum. There was also a significant association between the presence of *Chlamydia*-specific IgE antibodies in the serum and cultivable *Chlamydia* from BAL fluid, suggesting that the presence of viable, actively replicating organisms are more likely to induce pathogen-specific IgE production and could therefore lead to greater allergic type pathology. A limitation of the current study however, is that we were not able to assess individual BAL samples for the presence of *Chlamydia*-specific IgE because of limited sample volume and therefore have only qualitative data on BAL IgE from pools of 4–6 samples. However, we assessed individual serum samples that showed similar banding patterns as the pooled BAL samples. A second limitation is that our control cohort consisted of only 35 patients and we did not obtain BAL samples from these patients since this is a highly invasive procedure. These patients were not assessed clinically for any respiratory disease since they appeared healthy upon physical examination as part of a wellness checkup by a primary care physician. However, compared to the respiratory disease population, these healthy controls did not have *Chlamydia*-specific IgE antibodies in their sera, suggesting that this phenomenon is specific to a chronic, severe, respiratory disease.

Chlamydial antigens recognized by specific IgE antibodies were significantly different in the BAL and serum for *C. trachomatis* versus *C. pneumoniae*. It is not clear at the present time why these dramatic differences in antigen recognition exist. However, only 6 chlamydial antigens were recognized by either BAL or serum specific IgE in this patient cohort. The only obvious unique quality about these antigens is that they are surface exposed or secreted by the organism. We have assessed these proteins by utilizing reducing and non-reducing SDS-PAGE conditions with the same results. We confirmed the identity of the MOMP protein and the cLPS antigen using recombinant proteins but used the *Chlamydia* protein data base to match the most likely molecular weight for the other antigens. Further assessment of the patient cohort for the presence and level of total IgE confirmed that only 31% of patients who had *Chlamydia*-specific IgE had an overall elevated IgE level. This strongly suggests that patients who are assessed as non-atopic may nonetheless produce *Chlamydia*-specific IgE, which we hypothesize, could lead to airway pathology.

The primary biological function of IgE is to provide immunity against multicellular parasitic pathogens [36]. However, in developed countries with good sanitation where parasitic diseases are rare, IgE responses are most often directed against innocuous allergens resulting in type I allergic disease. Mouse-model studies have consistently demonstrated that inhalation of innocuous aerosolized protein antigens typically does not induce antigen-specific Th2 responses leading to IgE production [37]. However, it has been demonstrated that ongoing Th2 responses produced either by antigen challenge or infection during antigen inhalation can prevent the establishment of aerosol-induced IgE tolerance and lead instead to Th2 priming [38]. It is possible as suggested by Hollams *et al*[39], that upon bacterial infection Th2 cytokines may just be suppressing other pro-inflammatory cytokines, such as tumor necrosis factor-α and IL-1, as well as mediators such as prostaglandin, hence, counter-regulating the increased Th1 immune responses. However, since *C. pneumoniae* surface antigens have to be processed and presented by antigen presenting cells (APC) to activate T-cells and, thus, may not directly induce a Th2 response, this theory might not hold true in this case.

## Conclusions

*C. pneumoniae* has long been implicated in the inflammatory response experienced by chronic asthmatics; however, a specific mechanism of its involvement has remained elusive. While the role and regulation of IgE antibodies towards microbial antigens is far from being elucidated, results from the current study strongly suggest that this obligate intracellular pathogen can induce pathogen-specific IgE production and could therefore lead to mast cell degranulation and release of vasoactive agents. The presence of *Chlamydia*-specific IgE in the serum would suggest that the organisms play a direct role in asthma pathogenesis by continuous induction of IgE, since unlike most aeroallergens that a patient can avoid, the chlamydial organisms reside in the lower airways and are continuously secreting bacterial antigens. In addition, even if the organism is cleared by antibiotic use or natural immune responses, *Chlamydia* – specific IgE antibodies might still play an important role in the development of exaggerated airway responsiveness during subsequent subclinical or even asymptomatic infection.

## Competing interests

The authors declare that they have no competing interests.

## Authors’ contributions

WW and PS conceived of the study and participated in its design, coordination, supervision, data analysis and drafted and the manuscript. PS was responsible for patient selection and sample collection. KP performed the cultures and PCR as well as assisting in the data analysis and drafting/revision of the manuscript. EA performed PCR on some samples, developed and optimized the *Chlamydia*-specific Western blot method, performed data analysis and participated in the final manuscript review. KP and EA therefore share first author status. All authors read and approved the final manuscript.
